# Dietary phosphorus intake and its association with metabolic syndrome and its components: a cross-sectional analysis of the UK national diet and nutrition survey (NDNS)

**DOI:** 10.1007/s00394-025-03882-9

**Published:** 2026-02-12

**Authors:** R. E. Khoury, O. Obeid, M. Malla, A. Avery, S. Welham

**Affiliations:** 1 Division of Food, Nutrition & Dietetics, University of Nottingham, Sutton Bonington Campus, Loughborough, LE12 5RD UK; 2 Department of Nutrition and Food Sciences, American University of Beirut, Beirut, Lebanon; 3 Department of Epidemiology and Biostatistics, American University of Beirut, Beirut, Lebanon

**Keywords:** Phosphorus, Glycemia, Blood pressure, Dyslipidemia, Metabolic syndrome, Cross-sectional analysis, National diet and nutrition survey

## Abstract

**Purpose:**

Phosphorus plays a critical role in carbohydrate and energy metabolism, yet its relationship with metabolic health outcomes remains underexplored. This study aimed to investigate the association between dietary phosphorus intake and the prevalence of metabolic syndrome (MetS), as well as individual MetS components, using data from the UK National Diet and Nutrition Survey.

**Methods:**

Data from adults aged 19 years and older were analyzed. Dietary phosphorus intake was assessed using four-day food diaries. MetS was defined based on established clinical criteria. Logistic regression models evaluated the association between phosphorus intake quintiles and MetS occurrence, adjusting for demographic, anthropometric, dietary, and lifestyle factors. Associations between phosphorus intake and individual MetS components were examined based on both total phosphorus intake and phosphorus density.

**Results:**

Individuals in the highest phosphorus intake quintile (> 1509 mg/day) exhibited a 56% lower risk of MetS compared to those in the lowest quintile (OR = 0.44, *p*  = 0.0004). Higher phosphorus intake was associated with a decrease of 10.4 mg/dl in triglyceride levels from quintile 1 to quintile 5 (mean ± SD: 118.6 ± 87.5 vs. 108.2 ± 61.7, *p* =  0.002), as well as a 2.1 mmHg reduction in diastolic blood pressure (mean ± SD: 74.6 ± 11.1 vs. 72.5 ± 10.7, *p* = 0.001). Additionally, modest variations in HDL cholesterol and waist circumference were observed.

**Conclusion:**

Higher dietary phosphorus intake was associated with a lower risk of MetS and beneficial differences in certain MetS components, supporting a potential protective role of phosphorus in metabolic health.

**Supplementary Information:**

The online version contains supplementary material available at 10.1007/s00394-025-03882-9.

## Background

Phosphorus is an essential macromineral that plays a critical role in various physiological processes, including energy metabolism, cellular signaling, and skeletal health [1, 2]. While its function in bone mineralization is well established, emerging evidence suggests that its involvement extends beyond mineral balance to influence key metabolic pathways such as glucose regulation, lipid metabolism, insulin sensitivity, and energy expenditure [1, 3, 4].

In recent decades, shifts in eating behavior and the transition from traditional diets rich in complex carbohydrates toward dietary patterns characterized by higher intakes of refined sugars, simple carbohydrates, and ultra-processed foods have been strongly associated with the global increase in metabolic disorders, including insulin resistance, abdominal obesity, and hypertension [5–8]. These dietary changes have also contributed to a reduction in the intake of naturally occurring micronutrients, particularly phosphorus [9]. Although the use of phosphorus-containing additives as inorganic phosphorus sources is widespread in modern food processing, organic phosphorus derived from unprocessed food sources is thought to confer distinct and potentially beneficial metabolic effects [10–12]. Phosphorus is widely distributed across various foods, with the main dietary contributors being animal-based products such as milk, dairy, meat, poultry, fish, and eggs, followed by grain products, leavened baked goods, and legumes [13].

It plays a crucial role in a wide range of biochemical reactions, particularly those involved in carbohydrate metabolism and insulin signaling. It is required for the phosphorylation of glucose to glucose-6-phosphate —a key initial step for cellular glucose uptake and intracellular glucose trapping— which is central to maintaining glucose homeostasis [3]. Additionally, phosphorus is indispensable for the synthesis of adenosine triphosphate (ATP), the primary energy currency of all cells, highlighting its fundamental role in both postprandial energy metabolism and thermogenesis [4]. Postprandial hepatic ATP production has also been shown to influence appetite regulation by stimulating hepatic vagal afferent activity, which transmits information about the liver’s energy status to the central nervous system, thereby promoting satiety and modulating subsequent food intake [14]. Given these physiological roles, several human and animal studies have highlighted a potential role for phosphorus intake in modulating postprandial glycemia, lipid profiles, and diet-induced thermogenesis, supporting a broader contribution of this nutrient to metabolic health [15–24]. However, the relationship between habitual dietary phosphorus intake and the occurrence of metabolic syndrome remains poorly understood in population-based studies.

Metabolic syndrome (MetS) is characterized by a cluster of interconnected metabolic disturbances, including abdominal obesity, hyperglycemia, dyslipidemia, and elevated blood pressure, which collectively increase the risk of developing type 2 diabetes mellitus and cardiovascular disease [25, 26]. The global prevalence of MetS has risen substantially in parallel with modern dietary transitions and lifestyle changes. While the underlying mechanisms contributing to the development of MetS are multifactorial, disturbances in energy metabolism, insulin resistance, and nutrient intake have been identified as key contributors [25]. Given the involvement of phosphorus in energy metabolism, insulin signaling, and appetite regulation, the investigation of dietary phosphorus intake in relation to MetS represents an important step toward improving the understanding of nutrient-related determinants of metabolic health.

Despite its physiological importance, population-level studies examining habitual dietary phosphorus intake in relation to metabolic health remain limited. The Reference Nutrient Intake (RNI) for phosphorus in the United Kingdom (UK) is set at 550 mg per day for healthy adults [2], yet its potential association with MetS and its individual components has not been thoroughly investigated in large, nationally representative cohorts. Hence, the current study aimed, first, to assess the distribution of daily phosphorus intake, its dietary sources, and intake patterns among UK adults; second, to examine the association between dietary phosphorus intake and the occurrence of MetS; and third, to investigate the association between dietary phosphorus intake and the individual components of MetS, using data from the National Diet and Nutrition Survey (NDNS) Rolling Programme.

## Methods

### Data source

The NDNS Rolling Programme is a continuous cross-sectional survey designed to examine the dietary habits, food consumption, nutrient intakes and nutritional status of people aged 1.5 years and older, living in private households in the UK [26]. The survey, undertaken between years 2008 and 2018, is carried out in all four countries of the UK (England, Northern Ireland, Wales and Scotland), and is designed to be representative of the UK population. Pregnant and breastfeeding women were excluded from the survey due to their special nutritional needs. The survey includes a one-to-one interview, a 4-day estimated food diary, physical (anthropometric) and biochemical (blood and urine) measurements.

### Study design and population

This study is a secondary cross-sectional analysis of the NDNS dataset. Data from years 1–10 (2008–2018) were utilized for individuals aged 19 years and older.

**Fig.1:**
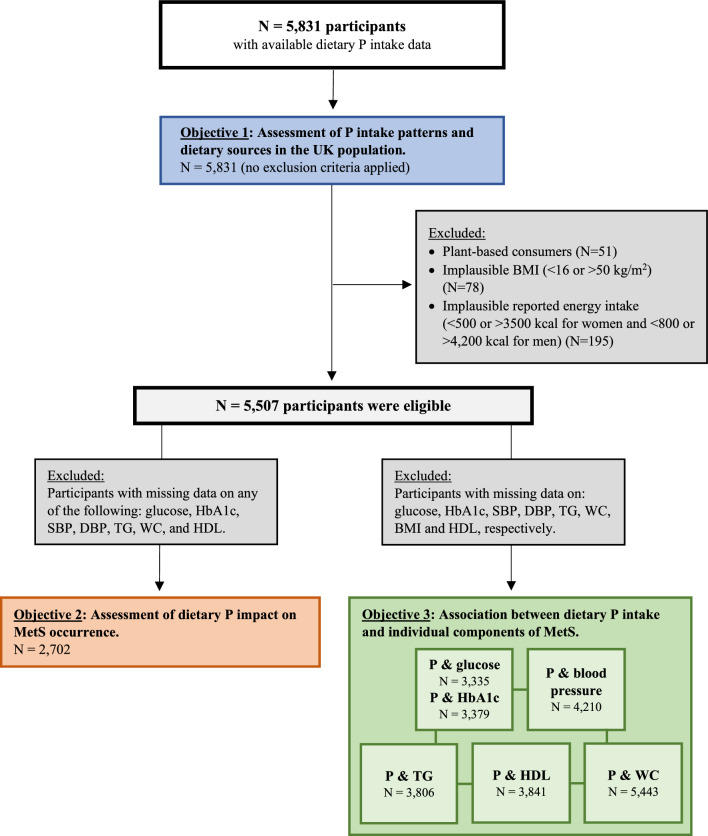
Flowchart of exclusion criteria and study population selection for each analysis. P, phosphorus; NDNS, National Diet and Nutritional Survey; UK, United Kingdom; BMI, body mass index; HbA1c, glycated hemoglobin; SBP, systolic blood pressure; DBP, diastolic blood pressure; TG, triglycerides; WC, waist circumference; BMI, body mass index; HDL, high-density lipoprotein; MetS, metabolic syndrome.

For the first objective, all individuals with available dietary phosphorus intake data were included in the analysis without any exclusions. This approach allowed for a comprehensive analysis of phosphorus consumption trends across different demographic groups, dietary patterns, and age categories. For the second and third objectives, a two-level inclusion process was implemented. The first level involved the exclusion of extreme outliers based on predefined criteria, while the second level applied additional filtering based on data availability specific to each MetS component. Furthermore, because Objectives 2 and 3 aimed to assess how dietary phosphorus intake and its postprandial availability relate to metabolic outcomes, individuals with minimal phosphorus absorption profiles were excluded. Specifically, those who identified as vegans were excluded due to their reliance on plant-based phosphorus sources, which predominantly comprise phytate. Phytate, a naturally occurring compound in plant foods, contains phosphate as part of its native structure. Humans lack the enzyme phytase and consequently, the phosphate contained within it is inaccessible unless plant foods are appropriately processed before consumption [13]. In contrast, vegetarians who consumed dairy and eggs were retained in the analysis, as these foods provide highly bioavailable phosphorus. A visual representation of the inclusion process is illustrated in Fig. 1.

### Dietary assessment

NDNS participants were asked to retain a record of everything eaten and drunk over a period of four consecutive days. Estimations of serving and portion sizes were self-described using standard household measures and/or weights from nutritional labels. Records were then reviewed by trained assessors during face-to-face visits, to ensure validity and precision of all the self-reported dietary information.

### Health markers

Blood sampling took place two to four months after completion of the 4-day food diary by qualified nurses. Blood samples were collected following an overnight fast of approximately 8 h for all adults, except for individuals with diabetes or those who declined to fast, who provided non-fasting blood samples instead. Venepuncture was schedule before midday, and all samples were processed within two hours of collection. Blood pressure was recorded using an Omron-HEM907 automated validated monitor. Insights on glycemic status were inferred from glucose and glycated hemoglobin (HbA1c) measured in blood samples. HbA1c was measured using high-performance liquid chromatography (HPLC) with a Tosoh Automated Glycohemoglobin Analyser. Plasma glucose concentrations were determined using Siemens Dimension clinical chemistry analyzers, following the manufacturer’s standard protocols. Total cholesterol, high-density lipoprotein (HDL) cholesterol, and triglycerides were measured enzymatically using Siemens Dimension clinical chemistry analyzers. Low-density lipoprotein (LDL) cholesterol was calculated using the Friedewald equation. Anthropometric measurements were conducted following standardized NDNS protocols. Weight was measured to the nearest 0.1 kg using calibrated digital scales. Height was measured to the nearest 0.1 cm using a stadiometer, with participants standing upright without shoes. Waist circumference was measured to the nearest 0.1 cm at the midpoint between the lower margin of the last palpable rib and the iliac crest, according to the WHO STEPwise approach to Surveillance (STEPS) protocol, using a non-stretchable tape measure. All measurements were performed by trained personnel to ensure accuracy and reproducibility. Body mass index (BMI) was calculated as weight in kilograms divided by the square of height in meters (kg/m^2^), using measured weight and height values.

### Phosphorus intake

Macro and micronutrient intake levels, including that of phosphorus, were available in the NDNS database and were deduced from participants’ food records using DINO (Diet In Nutrients Out), an integrated dietary recording and analysis system, and the UK Nutrient Databank. The UK Nutrient Databank is generated in accordance with food tables from ‘McCance and Widdowson’s Composition of Foods’, the Food Standards Agency’s food portion sizes, and data from manufacturers. All foods listed in the NDNS dataset were categorized into major food groups. Each food group’s contribution to daily phosphorus intake per individual was calculated separately. The contribution was then summed across the study population, divided over the sum of daily phosphorus intake, and grouped by gender. It should be noted that the NDNS database does not provide reliable differentiation between phosphorus originating from naturally occurring food sources and that from inorganic food additives or supplements, nor does it specify the chemical form of phosphorus present, which limits the precision of bioavailability estimates in this analysis.

### Classification of subjects with metabolic syndrome

According to the National Cholesterol Education Program Expert Panel (NCEP) and Adult Treatment Panel III (ATP III) criteria, an individual is classified as having MetS if they meet at least three of the following criteria: 1) elevated waist circumference: 88 cm or more for women, and 102 cm or more for men; 2) elevated blood pressure: 130 mm Hg or more for systolic blood pressure (SBP), or 85 mm Hg or more for diastolic blood pressure (DBP), or taking any antihypertensive drug treatments; 3) elevated triglyceride level: 150 mg/dl or more; 4) reduced HDL cholesterol level: 50 mg/dl or less for women, and 40 mg/dl or less for men; 5) elevated fasting blood sugar level: above 5.55 mmol/L, or taking any glucose-lowering medications [26].

### Covariates

Several covariates were included in the analysis to account for potential confounding, selected based on their established relevance in the literature and their potential impact on both phosphorus metabolism and MetS components. Demographic variables included age and sex to control for age-related metabolic variations and sex differences in dietary intake and MetS prevalence. The anthropometric variables included weight, height, and BMI. Lifestyle factors included smoking status and alcohol consumption. To account for individual variations in habitual energy intake and minimize the impact of misreporting, energy density adjustments were applied by calculating phosphorus intake per 1000 kcal, through the ratio of daily phosphorus intake to total caloric consumption. This approach complements total intake analysis and enables the evaluation of nutrient quality independent of total energy intake.

### Statistical analysis

Analyses were performed in *SPSS Statistics* (version 26) and *R* (version 4.2.2). Categorical data were presented as N (%). Continuous data were presented either as mean values with standard deviation or as median with 25th–75th percentiles. To explore the association between phosphorus intake and MetS, participants were divided into quintiles based on their phosphorus intake distribution. Each quintile represented 20% of the study population, ensuring equal group sizes and facilitating interpretation of dose–response patterns. Logistic regression models were used to examine the association between phosphorus intake and the risk of MetS. MetS was treated as a binary outcome variable. The analyses were conducted using Generalized Linear Models (GLM) with a binomial family and the logit link function. For all models, the significance of individual predictors was evaluated using Wald tests. The odds ratios (OR) and their 95% confidence intervals (CI) were computed for each predictor to quantify the magnitude of associations. Variance inflation factors (VIF) were assessed to check for multicollinearity among the predictors. Model fit was assessed using the Hosmer–Lemeshow goodness-of-fit test, with a non-significant p-value suggesting good model fit. Kruskal–Wallis test was used to assess differences in each MetS variable across phosphorus intake quintiles, followed by pairwise Wilcoxon rank-sum tests with Bonferroni correction. To adjust for potential confounders, analysis of covariance (ANCOVA) was performed using the aov() function. Type II ANOVA was conducted to further assess the significance of each predictor. Bonferroni correction was applied to account for multiple comparisons in post-hoc tests. P values of less than or equal to 0.05 were considered statistically significant throughout the study.

## Results

### Dietary phosphorus intake patterns and time trend analysis

The distribution of daily phosphorus intake across the whole population is presented in Table 1. The median dietary phosphorus intake was 1,193 mg/day, meeting the Reference Nutrient Intake (RNI) for healthy adults. Males consumed more dietary phosphorus than females (*p* < 0.0001), and age was a significant factor influencing intake in both sexes (*p* < 0.0001). Among males, phosphorus intake began to decline from the 40–49 years age group onward, whereas among females, intake generally increased with age until it declined in the 70–79 years age group (*p* < 0.0001).

**Table 1:** Daily phosphorus and energy intake stratified by sex and age

			Median (25th–75th percentiles) by sex				Median (25th–75th percentiles) across age groups														
N = 5,831							19–29		30–39		40–49		50–59		60–69		70–79		≥ 80		
Marker	Median	IQR	Sex	Median	IQR	*p*-value	Median	IQR	Median	IQR	Median	IQR	Median	IQR	Median	IQR	Median	IQR	Median	IQR	*p*-value
P intake (mg per day)	1193	961–1447	M	1345	1097–1610	< 0.0001***	1364	1098- 1641	1384	1172–1652	1358	1106- 1643	1364	1106- 1635	1342	1099- 1557	1292	1049- 1490	1178	965–1370	< 0.0001***
			F	1100	892–1322		1029	826–1256	1099	878–1312	1100	902–1339	1134	930–1355	1141	933–1341	1104	908–1306	977	792–1219	< 0.0001***
P intake (mg/1000 kcal per day)	677	602–773	M	692	592–741	< 0.0001***	632	555–704	656	594- 729	660	593–735	664	593–743	675	610–767	682	600–752	673	586- 771	< 0.0001***
			F	660	609–794		644	565- 730	665	601- 760	681	600- 785	716	623- 814	734	647- 824	734	644–849	703	622–792	< 0.0001***
Energy intake (kcal per day)	1726	1399–2105	M	2020	1654–2406	< 0.0001***	2175	1802–2540	2102	1776- 2514	2100	1674- 2498	2018	1681- 2405	1973	1614–2332	1847	1611–2218	1729	1487–1991	< 0.0001***
			F	1565	1285–1856		1584	1275- 1918	1629	1304- 1946	1587	1296- 1946	1582	1324- 1836	1529	1281- 1798	1517	1254–1781	1460	1184–1675	< 0.0001***

IQR, interquartile range; P, phosphorus; M, males; F, females; Asterisks indicate significance: *p  < 0.05; **p  < 0.01; ***p  < 0.001

**Fig.2:**
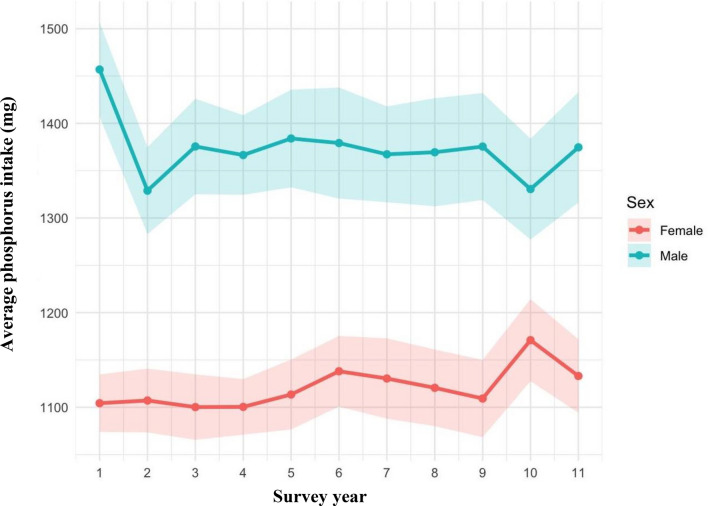
Average phosphorus intake (mg) among UK adults (19 + years), stratified by gender, across survey years 2008–2018. Shaded areas represent the 95% confidence interval around the mean.

The time-trend analysis revealed a gender specific relationship in phosphorus intake levels across the survey years 2008–2018, as illustrated in Fig. 2. Among males, dietary phosphorus declined from 1500 mg/day in year 1 (2008) to 1320 mg/day in year 10 (2018). In contrast, intake levels among females remained relatively stable over the survey period, with no significant changes observed.

### Contribution of major food groups

**Fig.3:**
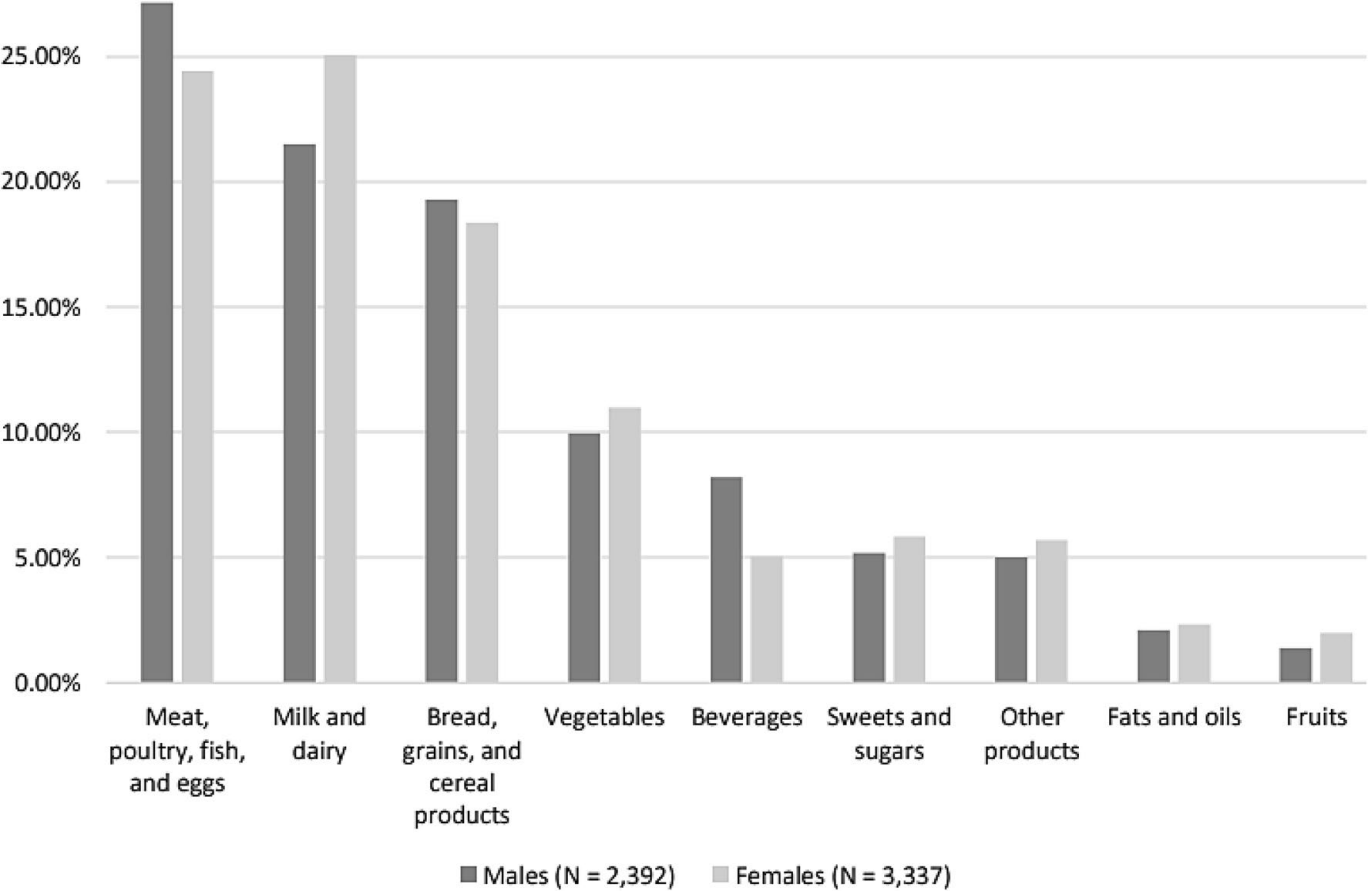
Contribution of major food groups to daily phosphorus intake

The contribution of major food groups to the daily intake of phosphorus, stratified by gender, is shown in Fig. 3. Animal-based foods were the highest contributors to daily phosphorus intake. Among males, the ‘meat, poultry, fish, and eggs’ subgroup was the main contributor to total phosphors intake (27.16%), followed by the ‘milk and dairy’ subgroup (21.51%). Alternatively, among females, the ‘milk and dairy’ subgroup was the main contributor to total phosphors intake (25.05%), followed by the ‘meat, poultry, fish, and eggs’ subgroup (24.42%). ‘Fats and oils’, ‘fruits’, and ‘dietary supplements’ were the lowest contributors to daily phosphorus intake in both males and females. Pairwise comparisons of mean phosphorus intake showed significant differences between each of the following main food groups, in both males and females: (i) meat, poultry, fish, and eggs; (ii) milk and dairy; (iii) bread, grains, and cereal products; (iv) vegetables; and (v) beverages, with the exception of mean phosphorus intake from (meat, poultry, fish, and eggs) and (milk and dairy) food groups among females, which appeared to be rather equivalent (Online Resource 1).

### Metabolic syndrome: occurrence and associated risk

Over the survey years 2008–2018, 681 participants (25.2%) met at least three criteria for MetS and were classified as having the condition. The characteristics of participants with and without MetS are presented in Table 2.

**Table 2:** Prevalence of MetS and its association with key variables

Variables N = 2702	No MetS N = 2,021 (74.8%)	MetS N = 681 (25.2%)	*p *-value
*Sex*			
Males	786 (70.6%)	328 (29.4%)	< 0.0001***
Females	1235 (77.8%)	353 (22.2%)	
*Age (years)*			
19–29	275 (94.2%)	17 (5.8%)	< 0.0001***
30–39	367 (84.6%)	67 (15.4%)	
40–49	452 (77.5%)	131 (22.5%)	
50–59	363 (68%)	171 (32%)	
60–69	298 (63.1%)	174 (36.9%)	
70–79	183 (67.5%)	88 (32.5%)	
> 79	83 (71.6%)	33 (28.4%)	
*BMI category (kg/m*^*2*^*)*			
< 18.5	19 (100%)	0 (0%)	< 0.0001***
18.5–24.9	838 (95.9%)	36 (4.1%)	
25.0–29.9	787 (74.5%)	270 (25.5%)	
30.0–39.9	353 (51.6%)	331 (48.4%)	
> 40.0	24 (35.3%)	44 (64.7%)	
*Smoking status*			
Non-smoker	1761 (75.3%)	577 (24.7%)	0.112
Smoker	260 (71.4%	104 (28.6%)	
*Drinking status*			
Non-drinker	707 (71.1%)	287 (28%)	< 0.0001***
Drinker	1314 (76.9%)	394 (23.1)	
*Dietary P intake*			
Daily P intake (mg/day)	1248 (371)	1,222 (363)	0.217
P density (mg/1000 kcal)	705 (146)	701 (153)	0.595
*Daily P intake quintiles (mg/day)*			
< 915	356 (71.1%)	145 (28.9%)	0.0732
915 – 1106	413 (76.2%)	129 (23.8%)	
1107 – 1294	413 (76.2%)	129 (23.8%)	
1295 – 1509	385 (72.5%)	146 (27.5%)	
> 1509	454 (77.5%)	132 (22.5%)	
Fasting blood glucose (mg/dl)	92.3 (16.0)	111.0 (30.0)	< 0.0001***
HbA1c (%)	5.5 (0.6)	6.0 (1.0)	< 0.0001***
Systolic blood pressure (mmHg)	122.4 (15.7)	136.4 (17.0)	< 0.0001***
Diastolic blood pressure (mmHg)	72.3 (10.4)	79.9 (11.3)	< 0.0001***
Triglycerides (mg/dl)	90.5 (46.1)	179.3 (88.8)	< 0.0001***
HDL cholesterol (mg/dl)	60.8 (17.0)	45.1 (13.6)	< 0.0001***
Waist circumference (cm)	89.2 (12.8)	104.6 (12.1)	< 0.0001***

MetS, metabolic syndrome; BMI, body mass index; P, phosphorus, HbA1c, glycated hemoglobin, HDL, high density lipoprotein

Categorical variables are presented as weighted percentage, while continuous data are presented as mean (standard deviation); Asterisks indicate significance: *p  < 0.05; **p  < 0.01; ***p  < 0.001

In both Models 1 and 2, logistic regression analysis indicated an inverse association between phosphorus intake and the odds of MetS (Table 3). In Model 1, adjusted for age, sex, and BMI, participants in the highest phosphorus intake quintile exhibited significantly lower odds of MetS compared with those in the lowest quintile (OR = 0.55; 95% CI: 0.40–0.76). A similar association was observed in Model 2, which additionally adjusted for energy intake, smoking status, and alcohol consumption, with the highest intake quintile demonstrating a significantly reduced likelihood of MetS (OR = 0.44; 95% CI: 0.28–0.70), representing a 56% lower risk compared with quintile 1.

**Table 3:** Logistic regression analysis of the association between dietary phosphorus intake and MetS risk

Phosphorus intake (mg/d)	N (= 2,702)	Crude OR (95 CI %)	*p*-value	Model 1 OR (95 CI %)	*p*-value	Model 2 OR (95 CI %)	*p*-value
QUINTILE 1 (< 915)	501	*Reference*	*–*	*Reference*	*–*	*Reference*	–
QUINTILE 2 (915 − 1,106)	542	0.77 (0.58, 1.01)	0.059	0.67 (0.48, 0.92)	0.013*	0.66 (0.47, 0.91)	0.014*
QUINTILE 3 (1,107–1,294)	542	0.77 (0.58, 1.01)	0.059	0.64 (0.46, 0.87)	0.005**	0.59 (0.41, 0.82)	0.002**
QUINTILE 4 (1,295–1,509)	531	0.93 (0.71, 1.22)	0.605	0.77 (0.56, 1.04)	0.096	0.70 (0.48, 1.01)	0.059
QUINTILE 5 (> 1,509)	586	0.71 (0.54, 0.94)	0.016*	0.55 (0.39, 0.76)	0.0003***	0.44 (0.28, 0.69)	0.0004***

OR, odds ratio; CI, confidence interval

Model 1: adjusted for age, sex, and body mass index; Hosmer–Lemeshow goodness-of-fit χ^2^ = 13.67, *p * = 0.091

Model 2: adjusted for age, sex, BMI, daily energy intake, smoking, and drinking status; Hosmer–Lemeshow goodness-of-fit χ^2^ = 14.37, *p * = 0.073

Asterisks indicate significance: *p  < 0.05; **p  < 0.01; ***p  < 0.001

Older age and male sex were significantly associated with increased odds of MetS in both models. BMI categories yielded notably elevated odds ratios with by wide confidence intervals, potentially reflecting sparse data or quasi-separation.

Both models demonstrated acceptable overall fit. The Hosmer–Lemeshow goodness-of-fit test indicated no evidence of poor fit in either model (Model 1: χ^2^ = 13.67, *p *= 0.091; Model 2: χ^2^ = 14.37, *p *= 0.073). Multicollinearity was not a concern, as all generalized variance inflation factors (GVIFs) were well below the commonly accepted threshold of 2.

### Dietary phosphorus and components of metabolic syndrome

The association between dietary phosphorus and the individual components of MetS was evaluated using two measures: total phosphorus intake and total energy-adjusted phosphorus intake (phosphorus density), defined as milligrams of phosphorus intake per 1000 kilocalories. For each measure, analyses were conducted using unadjusted models and models adjusted for age, sex, and BMI. The Spearman’s rank correlation coefficients between total phosphorus intake levels (mg/day) and components of MetS are presented in Online Resource 2.

#### Total phosphorus intake and metabolic syndrome components

In the adjusted model, higher phosphorus intake was significantly associated with differences in HDL cholesterol, triglyceride levels, and waist circumference (Table 4). HDL cholesterol levels were significantly higher in quintile 3 (58.1 mg/dl, SD = 17.6) compared to the quintile 1 (56.9 mg/dl, SD = 18.4), but significantly lower in quintile 5 (54.7 mg/dl, SD = 16.4) compared to quintile 1, suggesting a non-linear relationship. Waist circumference was significantly higher in quintile 4 (93.3 cm, SD = 13.9) compared to quintile 1 (91.7 cm, SD = 14.3). Additionally, triglyceride levels were significantly higher in the lowest phosphorus intake group (quintile 1) (119.0 mg/dl, SD = 81.9) compared to all other quintiles.

**Table 4:** Associations of total phosphorus intake quintiles with individual components of MetS

Total P intake (mg/day)	QUINTILE 1 (< 915)				QUINTILE 2 (915 – 1,106)				QUINTILE 3 (1,107 – 1,294)				QUINTILE 4 (1,295 – 1,509)				QUINTILE 5 (> 1,509)					
	Mean	SD	Med	IQR	Mean	SD	Med	IQR	Mean	SD	Med	IQR	Mean	SD	Med	IQR	Mean	SD	Med	IQR	*p-*value†	*p-*value‡
N = 3335	624				670				667				658				716					
FBG (mg/dl)	96.7	21.9	91.8	14.2	95.8	19.6	92.1	13.0	95.5	17.6	93.2	13.5	97.7	25.0	93.3	14.1	97.8	23.9	92.6	13.4	0.201	0.151
N = 3379	634				686				683				667				709					
HbA1c (%)	5.6	0.8	5.5	0.5	5.6	0.7	5.4	0.6	5.6	0.6	5.5	0.5	5.6	0.7	5.5	0.5	5.6	0.8	5.4	0.4	0.461	0.056
N = 4210	829				852				842				837				850					
SBP (mmHg)	125.4	18.7	122.5	24.5	125.3	18.4	123.0	24.3	125.8	16.7	124.0	22.0	126.5	17.1	124.5	21.5	126.9	15.6	125.8	19.5	0.005**	0.415
DBP (mmHg)	73.7	11.3	73.0	14.5	73.5	10.7	73.0	14.0	73.5	11.3	73.0	15.0	73.7	10.8	73.5	15.5	73.9	11.4	73.5	14.5	0.904	0.717
N = 3806	712				764				764				763				803					
TG (mg/dl)	119.0^a^	81.9	97.4	70.9	111.9^b^	69.0	88.6	62.0	107.9^b^	65.6	88.6	62.0	113.2^b^	74.8	97.4	70.9	116.6^b^	80.8	97.4	79.7	0.339	< 0.0001***
N = 3841	721				771				771				769				809					
HDL (mg/dl)	56.9^a^	18.4	54.1	22.8	57.3	17.5	54.1	22.4	58.1^b^	17.6	55.3	22.8	56.5	17.2	53.8	21.7	54.7^b^	16.4	52.6	21.3	0.004**	0.001**
N = 5443	1083				1081				1094				1083				1102					
WC (cm)	91.7^a^	14.3	90.5	19.2	92.8	14.9	92.4	21.9	93.0	14.4	91.7	20.4	93.3^b^	13.9	92.8	20.3	94.7	14.3	93.5	19.6	< 0.0001***	0.001**

P, phosphorus; SD, standard deviation; Med, median; IQR, interquartile range; FBG, fasting blood glucose; HbA1c, glycated hemoglobin; SBP, systolic blood pressure; DBP, diastolic blood pressure; TG, triglycerides; HDL, high density lipoprotein cholesterol; WC, waist circumference

^†^ Unadjusted p-values derived from the Kruskal–Wallis test to assess if each MetS component differs across total phosphorus quintiles

^‡^ *P *-values adjusted for age, sex, and BMI, derived from the Kruskal–Wallis test to assess if each MetS component differs across total phosphorus quintiles

Values with different superscripts (a, b, c) indicate statistically significant differences between phosphorus total intake quintiles based on Bonferroni-adjusted *p *-values from pairwise Wilcoxon rank-sum tests. Superscripts are not applied to unadjusted values. Pairwise comparisons were conducted only where the Kruskal–Wallis test indicated overall group differences. Bonferroni correction was used to adjust for multiple testing across all 10 pairwise comparisons; Asterisks indicate significance: *p  < 0.05; **p  < 0.01; ***p  < 0.001

#### Phosphorus density and metabolic syndrome components

Following adjustments for age, sex, and BMI, phosphorus density showed similar associations with triglyceride levels and waist circumference as observed with total phosphorus intake (Table 5). Triglyceride levels were significantly lower in quintile 5 (108.2 mg/dl, SD = 61.7) compared to quintile 1 (118.6 mg/dl, SD = 87.5), with a difference of 10.4 mg/dl (*p* = 0.002). Mean waist circumference decreased across increasing phosphorus intake quintiles: 92.5 cm in quintile 1 (SD = 14.6), 93.0 cm in quintiles 2 (SD = 14.2) and 3 (SD = 14.0), 93.5 cm in quintiles 4 (SD = 14.4) and 5 (SD = 14.8). Waist circumference was significantly lower in all lower phosphorus intake quintiles (quintiles 1 to 4) compared to the highest quintile (p < 0.05 for all).

**Table 5:** Associations of phosphorus density quintiles with individual components of MetS

Total P density (mg/1000 kcal)	QUINTILE 1 (< 585)				QUINTILE 2 (585 – 647)				QUINTILE 3 (648 – 711)				QUINTILE 4 (712 – 798)				QUINTILE 5 (> 799)					
	Mean	SD	Med	IQR	Mean	SD	Med	IQR	Mean	SD	Med	IQR	Mean	SD	Med	IQR	Mean	SD	Med	IQR	*p-*value†	*p-*value‡
N = 3335	653				648				690				672				672					
FBG (mg/dl)	95.8	19.4	92.6	14.1	95.9	17.0	93.0	13.0	97.4	22.8	92.9	13.9	96.9	23.1	91.7	13.0	97.4	25.4	92.6	14.0	0.598	0.714
N = 3379	677				658				699				674				671					
HbA1c (%)	5.6	0.7	5.4	0.4	5.5	0.6	5.5	0.5	5.6	0.8	5.5	0.5	5.6	0.8	5.5	0.5	5.7	0.8	5.5	0.5	0.128	0.627
N = 4210	793				834				857				849				877					
SBP (mmHg)	125.1	17.5	123.0	23.5	126.1	17.4	123.8	22.5	126.2	16.7	124.0	22.0	126.0	17.9	124.0	22.5	126.3	17.2	124.5	23.5	0.433	0.181
DBP (mmHg)	74.6^a^	11.1	73.0	15.0	74.5^a^	11.6	74.0	15.0	73.9^a^	10.8	73.0	15.0	73.8^a^	11.3	73.0	14.0	72.5^b^	10.7	72.5	13.5	0.008**	0.001**
N = 3806	749				748				776				767				766					
TG(mg/dl)	118.6^a^	87.5	97.4	70.9	114.8	76.5	97.4	79.7	114.9	68.7	97.4	70.9	112.0	76.9	97.4	62.0	108.2^b^	61.7	88.6	62.0	0.259	0.002**
N = 3841	756				756				783				774				772					
HDL (mg/dl)	55.7	17.8	52.6	22.4	55.7	16.6	53.2	21.1	55.9	17.1	53.0	20.9	57.8	17.9	55.1	23.6	58.2	17.6	55.7	22.4	0.002**	0.301
N = 5443	1087				1092				1094				1088				1082					
WC (cm)	92.5^a^	14.6	91.4	20.8	93.0^a^	14.2	92.4	20.5	93.0^a^	14.0	92.2	20.0	93.5^a^	14.4	93.1	20.1	93.5^b^	14.8	92.2	20.8	0.491	0.001**

P, phosphorus; SD, standard deviation; Med, median; IQR, interquartile range; FBG, fasting blood glucose; HbA1c, glycated hemoglobin; SBP, systolic blood pressure; DBP, diastolic blood pressure; TG, triglycerides; HDL, high density lipoprotein cholesterol; WC, waist circumference

^†^ Unadjusted *p *-values derived from the Kruskal–Wallis test to assess if each MetS component differs across phosphorus density quintiles

^‡^ *P*-values adjusted for age, sex, and BMI, derived from the Kruskal–Wallis test to assess if each MetS component differs across phosphorus density quintiles

Values with different superscripts (a, b, c) indicate statistically significant differences between phosphorus intake density quintiles based on Bonferroni-adjusted *p *-values from pairwise Wilcoxon rank-sum tests. Superscripts are not applied to unadjusted values. Pairwise comparisons were conducted only where the Kruskal–Wallis test indicated overall group differences. Bonferroni correction was used to adjust for multiple testing across all 10 pairwise comparisons; Asterisks indicate significance: *p  < 0.05; **p  < 0.01; ***p  < 0.001

The previously observed association between total phosphorus intake and HDL cholesterol was no longer evident in the energy-adjusted model. Additionally, DBP was inversely associated with phosphorus density, with participants in quintile 5 exhibiting a mean of 72.5 mmHg (SD = 10.7), 2.1 mmHg lower than those in quintile 1 (74.6 mmHg, SD = 11, *p * = 0.002). This association was not detected in the total phosphorus intake analysis. 

## Discussion

The present study found that higher phosphorus intake, particularly in the highest quintiles, was associated with a reduced risk of MetS. This association remained consistent across all models and appeared stronger after adjustment for demographic, lifestyle, and dietary factors, supporting a potential role for phosphorus in metabolic regulation. However, given the cross-sectional nature of the data, causality cannot be inferred, and the possibility of residual confounding or reverse causation, such as individuals with healthier metabolic profiles being more likely to consume phosphorus-rich foods, cannot be excluded. Additionally, the inability to distinguish between organic and inorganic sources of phosphorus, as well as between plant- and animal-based organic phosphorus, limits the precision of dietary phosphorus interpretation in this study. Nevertheless, these findings are broadly consistent with observational research reporting inverse associations between serum phosphate levels and components of MetS [25, 27–31].

Unlike many prior observational studies that primarily examine the association between total nutrient intake and MetS, this study incorporated both total phosphorus and total energy-adjusted phosphorus intake measures (phosphorus density), thereby providing a more nuanced understanding of phosphorus intake and its possible associations with metabolic health outcomes within the UK population.

When examining the individual components of MetS, total phosphorus intake revealed a non-linear association with HDL cholesterol, a direct association with waist circumference, and an inverse association with triglyceride levels. However, upon adjusting for energy intake, the associations with triglycerides and waist circumference persisted, an additional inverse association with DBP emerged, and the previously observed association with HDL cholesterol was no longer evident. The non-linear association observed between total phosphorus intake and HDL cholesterol was not replicated in the energy-adjusted analysis. This inconsistency may reflect model-specific variation or residual confounding by total energy intake. In the absence of reproducibility across models, the observed relationship cannot be considered causal or consistent.

Specifically, higher phosphorus density was associated with a 1 cm increase in waist circumference across quintiles, a reduction of approximately 2 mmHg in DBP, and a decrease of 10 mg/dl in triglyceride levels. Although these differences were statistically detectable in this large sample, they are modest at the individual level, suggesting greater relevance for population-level public health rather than individual clinical decision-making. Prior research has suggested that even modest reductions in blood pressure at the population level could lower the prevalence of hypertension by 17%, decrease stroke risk by 15%, and reduce the risk of coronary heart disease by 6% [32]. Similarly, the observed reduction in triglyceride levels may reflect potential lipid-related benefits of higher phosphorus intake.

The discrepancy between the findings derived from total phosphorus intake and phosphorus density highlights the importance of adjusting for energy intake in observational studies. While total phosphorus intake may provide insights into phosphorus consumption, the lack of energy adjustment can introduce confounding factors that obscure the observed associations between phosphorus and metabolic health. In contrast, phosphorus density, by accounting for total caloric intake, provides a more accurate reflection of the associations between phosphorus and metabolic outcomes [33]. This difference underscores the need for studies to consider phosphorus density, as relying solely on total phosphorus intake may lead to misleading conclusions and may not fully capture the nuances of the role of phosphorus in metabolic regulation.

Limited research has specifically examined the relationship between dietary phosphorus intake and individual components of MetS [34], especially since most existing cross-sectional studies have primarily focused on serum phosphate concentrations rather than dietary intake, making direct comparisons challenging [34, 35]. Nevertheless, the present results contribute valuable insights into possible links between dietary phosphorus and metabolic health, and align with broader trends observed in the literature. For example, the International Study of Macro- and Micronutrients and Blood Pressure (INTERMAP), a cross-sectional epidemiological study of 4,680 men and women from 17 population samples in Japan, China, the United Kingdom, and the United States, found that dietary phosphorus intake was inversely associated with blood pressure [36]. Estimated differences ranged from −1.9 to −4.2 mmHg for systolic blood pressure and −1.2 to −2.4 mmHg for diastolic blood pressure [36], consistent with the DBP results observed in the present study. Based on these outcomes, the INTERMAP study reinforced the importance of increasing phosphorus and mineral intake as part of dietary recommendations to support healthier eating patterns and potentially influence blood pressure [36]. Similarly, results from the present analysis align with findings from the Atherosclerosis Risk in Communities (ARIC) and Multi-Ethnic Study of Atherosclerosis (MESA) studies, which reported that higher phosphorus intake was associated with lower systolic and diastolic blood pressure levels [37]. Notably, the ARIC study observed a reduction of 2.0 mmHg (95% CI: −3.6 to −0.5; *p * = 0.01) between individuals in the lowest and highest intake categories [37], a finding comparable to the reduction observed between the first and fifth quintiles in the current study.

Although research on phosphate metabolism in dyslipidemia remains limited, the majority of available evidence, primarily derived from animal studies, showed consistent findings. Grundman et al. observed that mice fed a high-phosphorus diet had lower triglyceride levels and non-esterified cholesterol compared to those fed an adequate phosphorus diet [38]. Another research work by Abuduli et al. reported that rats on a high-phosphate diet showed reduced visceral fat accumulation and lower non-esterified fatty acids [16]. Despite the promising findings from various studies, the relationship between phosphate metabolism and dyslipidemia in humans remains inconclusive.

Regarding glycemia, while no significant association was observed between phosphorus intake (whether total or density-adjusted) and glucose or HbA1c in the present study, previous research reported differing outcomes. For instance, a recent nationwide cohort study in China observed a U-shaped association between dietary phosphorus intake and new-onset diabetes, with the lowest risk observed at intakes between 905 and 975 mg/day [39]. Similarly, the large prospective E3N French cohort study reported that phosphorus intakes exceeding 1477 mg/day were linked with an increased risk of type 2 diabetes [40].

Beyond glycemia, phosphorus intake has also been studied in relation to broader cardiovascular outcomes. Although experimental evidence provides biological plausibility for a link between excessive dietary phosphorus and cardiovascular disease (CVD) —mainly through mechanisms involving elevations in serum phosphate concentrations, vascular calcification, and endothelial dysfunction—findings from epidemiological studies remain inconsistent. One possible explanation is the relatively modest impact of dietary intake on serum phosphorus levels in individuals with normal kidney function [41]. For example, a large U.S. cohort of 15,513 participants from the Third National Health and Nutrition Examination Survey (NHANES III), each 500 mg/day increase in phosphorus intake was associated with only a 0.03 mg/dl rise in serum phosphorus, suggesting that phosphorus homeostasis is tightly regulated despite wide variations in intake and that such small increases may not substantially influence CVD risk [42].

Consistent with this uncertainty, the relationship between dietary phosphorus intake and direct cardiovascular outcomes also remains unclear. Several large cohort studies have reported inverse associations between phosphorus intake and blood pressure or hypertension risk, including the INTERMAP, ARIC, and MESA studies, which suggest a potential protective role [36, 37]. Similarly, among individuals with chronic kidney disease—who are typically more susceptible to phosphorus overload—higher phosphorus intake was not independently associated with increased mortality after adjusting for confounders [43]. In addition, a very recent study by Yang et al. found that higher dietary phosphorus intake was associated with lower cardiovascular mortality (HR: 0.43; 95% CI: 0.22–0.85 for the highest vs. lowest quartile; p for trend = 0.018) [44]. Another study using data from the Osteoporotic Fractures in Men (MrOS) cohort found no association between urinary phosphorus excretion (used as a proxy for intake) and CVD or all-cause mortality [45].

On the other hand, a few studies have reported potential harm. Elevated phosphorus intake has been linked to greater left ventricular mass in women, raising concerns about cardiovascular remodeling [46]. In addition, Chang et al. observed that higher dietary phosphorus intake was associated with increased cardiovascular and all-cause mortality in a healthy U.S. population [47]. Collectively, these findings highlight the conflicting nature of current cohort evidence, which does not consistently support a direct adverse relationship between phosphorus intake and cardiovascular endpoints. In the present analysis, higher phosphorus intake was associated with lower diastolic blood pressure and triglyceride levels. These findings may indicate potential cardiometabolic benefits and are broadly consistent with some earlier cohort studies. However, the relationship between dietary phosphorus and cardiovascular health appears complex and likely varies depending on dietary context, phosphorus source, and population characteristics.

Importantly, evidence from human populations remains limited, with key challenges including variability in phosphorus bioavailability, inaccuracies in dietary assessment methods, and potential confounding from other nutrients. As such, the present findings should be interpreted as hypothesis-generating and viewed within the context of these broader data limitations. In addition to these general challenges, the present study also faces specific limitations that warrant consideration. First, the NDNS relies on self-reported dietary intake data, introducing potential reporting bias and inaccuracies, particularly regarding daily fluctuations in phosphorus intake. This limitation is further compounded by the use of a four-day consecutive food diary, which, although widely accepted for detailed dietary assessment, may be affected by reactivity bias; participants aware of being monitored might alter their usual eating habits, potentially impacting data accuracy.

Additionally, the inability to distinguish between organic phosphorus from natural foods and inorganic phosphorus from additives and supplements is a major limitation, as these forms differ in bioavailability and metabolic effects. This issue is exacerbated by the broad categorization of food intake in the NDNS, which restricts accurate classification of phosphorus sources, particularly in composite foods such as salads, burgers, and mixed dishes − a challenge underscored by the European Food Safety Authority’s (EFSA) call for surrogate markers to improve the assessment of dietary phosphorus intake in large-scale population studies [2]. Moreover, the limited number of exclusive plant-based consumers prevented direct comparison between phosphorus intake from plant- and animal-based diets. Another methodological limitation pertains to the time lag between dietary recording and biomarker assessment. Blood samples for glucose, HbA1c, and lipid profiles were obtained two to four months after dietary data were recorded, which could attenuate observed associations, particularly for glycemia and waist circumference. While HbA1c is widely used for assessing glycemic control, it can be influenced by factors such as smoking, anemia, hemolysis, and infections, introducing additional variability [48]. Importantly, the lack of biochemical data on serum phosphorus precludes direct correlation between dietary intake and circulating phosphorus levels, limiting the scope of the findings. Furthermore, absence of information regarding medication use, such as phosphate binders or diuretics that could affect phosphorus metabolism constitutes another potential confounder.

Building on the limitations of the current study, future research is warranted to refine dietary phosphorus assessment methods through more detailed food coding systems and the incorporation of biomarker-based validation techniques. Integrating both dietary and biochemical data, while accounting for phosphorus sources, energy intake, and phosphorus density, will be essential to capture more nuanced associations. Additionally, longitudinal and interventional studies are needed to clarify potential causal relationships and uncover the underlying physiological mechanisms by which phosphorus may influence metabolic health. Such approaches are essential for informing evidence-based dietary guidelines aimed at the prevention and management of MetS.

## Conclusion

In this cross-sectional analysis of UK adults, higher dietary phosphorus intake was associated with improvements in metabolic markers, particularly lower DBP and triglyceride levels, as well as a reduced risk of MetS. Although these associations were statistically significant, the absolute differences in DBP and triglycerides are modest at the individual level, indicating that the observed effects are likely of greater relevance at the population level rather than for individual clinical decision-making.

Associations were more pronounced when phosphorus intake was expressed relative to total energy intake (phosphorus density), underscoring the importance of adjusting for caloric intake in nutritional epidemiology. These findings highlight the potential influence of dietary phosphorus on cardiometabolic health and support the consideration of phosphorus intake in public health strategies aimed at MetS prevention.

Given the observational design, causality cannot be inferred. Future research integrating both detailed dietary assessment and biochemical measurements of phosphorus, alongside longitudinal or interventional study designs, will be necessary to elucidate the underlying physiological mechanisms and clarify the role of phosphorus in metabolic regulation. 

## Supplementary Information

Below is the link to the electronic supplementary material.

Supplementary file1 (PDF 24 kb)

Supplementary file2 (PDF 215 kb)

## Data Availability

All data were taken from the National Diet and Nutrition Survey. These data are available from the Uk Data Service.

## References

[1] Raina R, Guarav G, Sidharth S et al (2012) Phosphorus metabolism. J Clin Nephrol Ther S003–008.

[2] EFSA NDA Panel (EFSA Panel on Dietetic Products, Nutrition and Allergies) (2015) Scientific opinion on dietary reference values for phosphorus. EFSA J 13 4185. 10.2903/j.efsa.2015.4185 10.2903/j.efsa.2015.4185 PMC13151608 42109813

[3] Bouché C, Serdy S, Kahn RC et al (2004) The cellular fate of glucose and its relevance in type 2 diabetes. Endocr Rev 25:807–830 15466941 10.1210/er.2003-0026

[4] Bonora M, Patergnani S, Rimessi A et al (2012) ATP synthesis and storage. Purinergic Signal 8:343–357 22528680 10.1007/s11302-012-9305-8 PMC3360099

[5] Popkin B (2006) Global nutrition dynamics: the world is shifting rapidly toward a diet linked with noncommunicable diseases. Am J Clin Nutr 84:289–298 16895874 10.1093/ajcn/84.1.289

[6] Lin X, Xu Y, Pan X et al (2020) Global, regional, and national burden and trend of diabetes in 195 countries and territories: an analysis from 1990 to 2025. Sci Rep 10:14790 32901098 10.1038/s41598-020-71908-9 PMC7478957

[7] Malik V, Willett W, Hu F et al (2013) Global obesity: trends, risk factors and policy implications. Nat Rev Endocrinol 9:13–27 23165161 10.1038/nrendo.2012.199

[8] Saklayen M (2018) The global epidemic of the metabolic syndrome. Curr Hypertens Rep 20:12 29480368 10.1007/s11906-018-0812-z PMC5866840

[9] Kant A (2000) Consumption of energy-dense, nutrient-poor foods by adult Americans: nutritional and health implications. The third national health and nutrition examination survey, 1988–1994. Am J Clin Nutr 72:929–936 11010933 10.1093/ajcn/72.4.929

[10] Ratsma D, Muller M, Koedam M et al (2024) Organic phosphate but not inorganic phosphate regulates Fgf23 expression through MAPK and TGF-ꞵ signaling. iScience. 10.1016/j.isci.2024.109625 38883842 10.1016/j.isci.2024.109625 PMC11178987

[11] Kawamura H, Tanaka S, Ota Y et al (2018) Dietary intake of inorganic phosphorus has a stronger influence on vascular-endothelium function than organic phosphorus. J Clin Biochem Nutr 62(2):167–173 29610557 10.3164/jcbn.17-97 PMC5874240

[12] Calvo MS, Uribarri J (2013) Public health impact of dietary phosphorus excess on bone and cardiovascular health in the general population. Am J Clin Nutr 98(1):6–15 23719553 10.3945/ajcn.112.053934

[13] Calvo M, Uribarri J (2013) Contributions to total phosphorus intake: all sources considered. Semin Dial 26(1):54–61 23278245 10.1111/sdi.12042

[14] Friedman M (2007) Obesity and the hepatic control of feeding behavior. Drug News Perspect 20:573–578 18176662 10.1358/dnp.2007.20.9.1162243

[15] Eller P, Eller K, Kirsch AH et al (2011) A murine model of phosphate nephropathy. Am J Pathol 175:1999–2006 10.1016/j.ajpath.2011.01.024 PMC3081192 21514417

[16] Abuduli M, Ohminami H, Otani T et al (2016) Effects of dietary phosphate on glucose and lipid metabolism. Am J Physiol Endocrinol Metab 310:E526–E538 26786774 10.1152/ajpendo.00234.2015

[17] Ellam T, Wilkie M, Chamberlain J et al (2011) Dietary phosphate modulates atherogenesis and insulin resistance in apolipoprotein E knockout mice. Arterioscler Thromb Vasc Biol 31:1988–1990 21636807 10.1161/ATVBAHA.111.231001

[18] Dwaib H, Ajouz G, AlZaim I, Rafeh R et al (2021) Phosphorus supplementation mitigates perivascular adipose inflammation-induced cardiovascular consequences in early metabolic impairment. Am Heart J 10(24):e023227 10.1161/JAHA.121.023227 PMC9075232 34873915

[19] Haglin L, Lindblad A, Bygren L (2001) Hypophosphataemia in the metabolic syndrome. Gender differences in body weight and blood glucose. Eur J Clin Nutr 55(6):493–498 11423926 10.1038/sj.ejcn.1601209

[20] Haap M, Heller E, Thamer C et al (2006) Association of serum phosphate levels with glucose tolerance, insulin sensitivity and insulin secretion in non-diabetic subjects. Eur J Clin Nutr 60:734–739 16391583 10.1038/sj.ejcn.1602375

[21] Akter S, Eguchi M, Kochi T, Kabe I, Nanri A, Mizoue T (2020) Association of serum calcium and phosphate concentrations with glucose metabolism markers: the Furukawa Nutrition and Health Study. Nutrients 12(8):2344 32764504 10.3390/nu12082344 PMC7468836

[22] Nowicki M, Fliser D, Fode P et al (1996) Changes in plasma phosphate levels influence insulin sensitivity under euglycemic conditions. J Clin Endocrinol Metab 81(1):156–159 8550745 10.1210/jcem.81.1.8550745

[23] Khattab M, Abi-Rashed C, Ghattas H et al (2015) Phosphorus ingestion improves oral glucose tolerance of healthy male subjects: a crossover experiment. Nutr J 14:112 26514124 10.1186/s12937-015-0101-5 PMC4627612

[24] Patti A, Al-Rasadi K, Giglio R et al (2018) Natural approaches in metabolic syndrome management. Arch Med Sci 14:422–441 29593818 10.5114/aoms.2017.68717 PMC5868676

[25] Kalaitzidis R, Tsimihodimos V, Bairaktari E et al (2005) Disturbances of phosphate metabolism: another feature of metabolic syndrome. Am J Kidney Dis 45:851–858 15861350 10.1053/j.ajkd.2005.01.005

[26] Program NCE (2002) Third report of the National Cholesterol Education Program (NCEP) expert panel on detection, evaluation, and treatment of high blood cholesterol in adults. Circulation 106(25):3143–3421 12485966

[27] Vyssoulis G, Karpanou E, Tzamou V et al (2010) Serum phosphate in white-coat hypertensive patients: focus on dipping status and metabolic syndrome. Hypertens Res 33(8):825–830 20505672 10.1038/hr.2010.86

[28] Gudmundsdottir H, Strand A, Kjeldsen S et al (2008) Serum phosphate, blood pressure, and the metabolic syndrome. J Clin Hypertens 10(11):814–821 10.1111/j.1751-7176.2008.00032.x PMC8673415 19128269

[29] Stoian M, Stoica V (2014) The role of disturbances of phosphate metabolism in metabolic syndrome. Maedica 9(3):255–260 25705287 PMC4305993

[30] Ghanei L, Ziaee A, Rostami P et al (2015) Association of serum 25-hydroxyvitamin d levels and vitamin D dietary intake with metabolic syndrome: a case control study. J res Health Sci 15(1):32–36 25821023

[31] Park W, Kim B, Lee J et al (2009) Serum phosphate levels and the risk of cardiovascular disease and metabolic syndrome: a double-edged sword. Diabetes Res Clin Pract 83(1):119–125 19101054 10.1016/j.diabres.2008.08.018

[32] Cook N, Cohen J, Hebert P et al (1995) Implications of small reductions in diastolic blood pressure for primary prevention. Arch Intern Med 155(7):701–709 7695458

[33] Rhee J, Cho E, Willett W (2014) Energy adjustment of nutrient intakes is preferable to adjustment using body weight and physical activity in epidemiological analyses. Public Health Nutr 17(5):1054–1060 23701939 10.1017/S1368980013001390 PMC3884063

[34] Wong S (2022) A review of current evidence on the relationship between phosphate metabolism and metabolic syndrome. Nutrients 14(21):4525 36364791 10.3390/nu14214525 PMC9656201

[35] Harvard T.H. Chan School of Public Health. (accessed 2025) Phosphorus. The Nutrition Source. https://nutritionsource.hsph.harvard.edu/phosphorus/

[36] Elliott P, Kesteloot H, Appel L et al (2008) Dietary phosphorus and blood pressure: international study of macro- and micro-nutrients and blood pressure. Hypertension 51(3):669–675 18250363 10.1161/HYPERTENSIONAHA.107.103747 PMC6556767

[37] Alonso A, Nettleton J, Ix J et al (2010) Dietary phosphorus, blood pressure, and incidence of hypertension in the atherosclerosis risk in communities study and the multi-ethnic study of atherosclerosis. Hypertension 55(3):776–784 20083730 10.1161/HYPERTENSIONAHA.109.143461 PMC2825283

[38] Grundmann S, Schutkowski A, Berger C et al (2020) High-phosphorus diets reduce aortic lesions and cardiomyocyte size and modify lipid metabolism in LDL receptor knockout mice. Sci Rep. 10.1038/s41598-020-77509-w 33247205 10.1038/s41598-020-77509-w PMC7695849

[39] Wu Q, Ye Z, Zhang Y et al (2023) A U-shaped association between dietary phosphorus intake and new-onset diabetes: a nationwide cohort study in China. Nutr Metab Cardiovasc Dis 33(10):1932–1940 37482482 10.1016/j.numecd.2023.03.002

[40] Mancini F, Affret A, Dow C et al (2018) High dietary phosphorus intake is associated with an increased risk of type 2 diabetes in the large prospective E3N cohort study. Clin Nutr 37:1625–1630 28818343 10.1016/j.clnu.2017.07.025

[41] Gutiérrez M (2013) The connection between dietary phosphorus, cardiovascular disease, and mortality: where we stand and what we need to know. Adv Nutr 4(6):723–729 24228204 10.3945/an.113.004812 PMC3823521

[42] Boer H, Rue C, Kestenbaum B (2009) Serum phosphorus concentrations in the third National Health and Nutrition Examination Survey (NHANES III). Am J Kidney Dis 53(3):399–407 18992979 10.1053/j.ajkd.2008.07.036 PMC3046032

[43] Murtaugh A, Filipowicz R, Baird B et al (2012) Dietary phosphorus intake and mortality in moderate chronic kidney disease: NHANES III. Nephrol Dial Transplant 27(3):990–996 21810769 10.1093/ndt/gfr367 PMC3350356

[44] Yang S, Chen H, Xie C et al (2025) Protective effect of dietary phosphorus intake on cardiovascular mortality in asthma: evidence from NHANES 1999-2018. Front Nutr 12:1533514 40070480 10.3389/fnut.2025.1533514 PMC11893399

[45] Dominguez J, Kestenbaum B, Chonchol M et al (2013) Relationships between serum and urine phosphorus with all-cause and cardiovascular mortality: the Osteoporotic Fractures in Men (MrOS) *Am*. J Kidney Dis 61(4):555–563 10.1053/j.ajkd.2012.11.033 PMC3815620 23261120

[46] Saab G, Whooley M, Schiller N et al (2010) Association of serum phosphorus with left ventricular mass in men and women with stable cardiovascular disease: data from the Heart and Soul Study. Am J Kidney Dis 56(3):496–505 20580478 10.1053/j.ajkd.2010.03.030 PMC2926276

[47] Chang A, Lazo M, Appel L et al (2014) High dietary phosphorus intake is associated with all-cause mortality: results from NHANES III. Am J Clin Nutr 99(2):320–327 24225358 10.3945/ajcn.113.073148 PMC3893724

[48] Bonora E, Tuomilehto J (2011) The pros and cons of diagnosing diabetes with A1C. Diabetes Care 34(2):184–190 21525453 10.2337/dc11-s216 PMC3632159

[49] Public Health England (2018) National Diet and Nutrition Survey results from years 7 and 8 (combined) of the rolling programme (2014/2015–2015/2016).

