# Supplementary material for: Dietary phosphorus intake and its association with metabolic syndrome and its components: a cross-sectional analysis of the UK national diet and nutrition survey (NDNS)
Source: Eur J Nutr. 2026 Feb 12;65(2):48. doi: 10.1007/s00394-025-03882-9 (PMC12901275; doi:10.1007/s00394-025-03882-9)
Supplement: Supplementary file 1 — Supplementary file1 (PDF 24 kb) [file 394_2025_3882_MOESM1_ESM.pdf]

Dietary Phosphorus Intake and Its Association with Metabolic Syndrome and Its Components: a Cross-Sectional Analysis of the UK National Diet and Nutrition Survey (NDNS)

EUROPEAN JOURNAL OF NUTRITION

[1]R.E. Khoury, [2]O. Obeid, [3]M. Malla, [1]A. Avery, and [1]S. Welham

[1]Division of Food, Nutrition & Dietetics, University of Nottingham, United Kingdom
[2]Department of Nutrition and Food Sciences, American University of Beirut, Lebanon
[3]Department of Epidemiology and Biostatistics, American University of Beirut, Lebanon

Corresponding author: Simon Welham
Simon.welham@nottingham.ac.uk

Online resource 1. Pairwise comparisons of mean phosphorus intake (mg) from main food groups.

Sex	Food group 1	Food group 2	Mean 1[a]	Mean 2[a]	p-value
**Males N=2,442**	Beverages	Bread, grains, and cereal products	110.12	262.42	**<0.0001***
	Beverages	Meat, poultry, fish, and eggs	110.12	379.92	**<0.0001***
	Beverages	Milk and dairy	110.12	292.00	**<0.0001***
	Beverages	Vegetables	110.12	82.37	**<0.0001***
	Bread, grains, and cereal products	Meat, poultry, fish, and eggs	262.42	379.92	**<0.0001***
	Bread, grains, and cereal products	Milk and dairy	262.42	292.00	**<0.0001***
	Bread, grains, and cereal products	Vegetables	262.42	82.37	**<0.0001***
	Meat, poultry, fish, and eggs	Milk and dairy	379.92	292.00	**<0.0001***
	Meat, poultry, fish, and eggs	Vegetables	379.92	82.37	**<0.0001***
	Milk and dairy	Vegetables	292.00	82.37	**<0.0001***
**Females N=3,389**	Beverages	Bread, grains, and cereal products	56.28	206.95	**<0.0001***
	Beverages	Meat, poultry, fish, and eggs	56.28	275.10	**<0.0001***
	Beverages	Milk and dairy	56.28	274.17	**<0.0001***
	Beverages	Vegetables	56.28	79.34	**<0.0001***
	Bread, grains, and cereal products	Meat, poultry, fish, and eggs	206.95	275.10	**<0.0001***
	Bread, grains, and cereal products	Milk and dairy	206.95	274.17	**<0.0001***
	Bread, grains, and cereal products	Vegetables	206.95	79.34	**<0.0001***
	Meat, poultry, fish, and eggs	Milk and dairy	275.10	274.17	**0.17**
	Meat, poultry, fish, and eggs	Vegetables	275.10	79.34	**<0.0001***
	Milk and dairy	Vegetables	274.17	79.34	**<0.0001***

[a] Mean 1 and Mean 2 represent the average phosphorus intake (mg/day) from Food group 1 and Food group 2, respectively.

*P-values < 0.05 denote statistical significance.
