# Supplementary material for: Dietary phosphorus intake and its association with metabolic syndrome and its components: a cross-sectional analysis of the UK national diet and nutrition survey (NDNS)
Source: Eur J Nutr. 2026 Feb 12;65(2):48. doi: 10.1007/s00394-025-03882-9 (PMC12901275; doi:10.1007/s00394-025-03882-9)
Supplement: Supplementary file 2 — Supplementary file2 (PDF 215 kb) [file 394_2025_3882_MOESM2_ESM.pdf]

Dietary Phosphorus Intake and Its Association with Metabolic Syndrome and Its Components: a Cross-Sectional Analysis of the UK National Diet and Nutrition Survey (NDNS)

EUROPEAN JOURNAL OF NUTRITION

[1]R.E. Khoury, [2]O. Obeid, [3]M. Malla, [1]A. Avery, and [1]S. Welham

[1]Division of Food, Nutrition & Dietetics, University of Nottingham, United Kingdom
[2]Department of Nutrition and Food Sciences, American University of Beirut, Lebanon
[3]Department of Epidemiology and Biostatistics, American University of Beirut, Lebanon

Corresponding author: Simon Welham
Simon.welham@nottingham.ac.uk

Online resource 2. Correlation between dietary phosphorus and the individual components of metabolic syndrome.

	Variable	N	Spearman's rho[a]	*p*-value
**Total phosphorus intake** (mg/day)	FBG	3,335	0.0406	**0.0190***
	HbA1c	3,379	-0.0174	0.3121
	SBP	4,210	0.069	**0.0000***
	DBP	4,210	0.0176	0.2542
	TG	3,806	-0.0017	0.9156
	HDL	3,841	-0.0348	**0.0309***
	WC	5,443	0.0688	**0.0000***
**Phosphorus density** (mg/1000kcal)	FBG	3,335	-0.0059	0.7316
	HbA1c	3,379	0.0488	**0.0045***
	SBP	4,210	0.0227	0.1408
	DBP	4,210	-0.0327	**0.0341***
	TG	3,806	-0.0231	0.1548
	HDL	3,841	0.063	**0.0001***
	WC	5,443	0.0223	0.0999

BMI, body mass index; FBG, fasting blood glucose; HbA1c, glycated hemoglobin; SBP, systolic blood pressure; DBP, diastolic blood pressure; TG, triglycerides; HDL, high density lipoprotein cholesterol; WC, waist circumference.
N represents the sample size for each metabolic syndrome component individually, based on data availability.
[a] Spearman's rho represents the correlation coefficient strength and direction of the relationship. Positive values indicate a direct relationship, and negative values indicate an inverse relationship.
*P-values < 0.05 denote statistical significance.
